# Effects of Aquatic Plants on Water Quality, Microbial Community, and Fish Behaviors in Newly Established Betta Aquaria

**DOI:** 10.3390/ani16020247

**Published:** 2026-01-14

**Authors:** Yidan Xu, Lixia Li, Yuting Chen, Yue Zhang, Tianyu Niu, Puyi Huang, Longhui Chai

**Affiliations:** College of Wildlife and Protected Area, Northeast Forestry University, Harbin 150040, China; 18340397657@163.com (Y.X.); lilixia0909@163.com (L.L.); m15167236817@163.com (Y.C.); 15040412694@163.com (Y.Z.); hireath_01@163.com (T.N.); chailonghui@126.com (L.C.)

**Keywords:** *Betta splendens*, *Sagittaria subulata*, *Alternanthera reineckii*, *Wolffia globosa*, ammonia nitrogen, behavioral evaluation, water microbiota, well-being

## Abstract

Maintaining water quality and fish well-being in newly established, small, unfiltered betta (*Betta splendens*) aquaria is a significant challenge. To improve betta fish breeding and welfare, this study set up four groups: the *Sagittaria subulata* (*S.su*) group, the *Alternanthera reineckii* (*A.re*) group, the *Wolffia globosa* (*W.gl*) group, and the plant-free (CG) group. We evaluated the effects of aquatic plants on water quality, fish behavior, and microbial community in newly established tanks over 25 days. The results demonstrated that both the dissolved oxygen (DO) and potential of hydrogen (pH) decreased with the experimental duration, while ammonia nitrogen (NH_3_-N) increased over time in all groups. Compared to the CG group, all aquatic plants significantly reduced the NH_3_-N accumulation. The *S.su* group exhibited the lowest mean NH_3_-N concentration of only 0.14 mg·L^−1^, which was considerably lower than that of the other groups (*p* < 0.05). The behavioral analysis revealed that, during the 25-day randomized monitoring period, bettas in the *S.su* group exhibited the lowest surface breathing, with an average of only 0.36 events per 5 min, which was significantly lower than that of the CG group (*p* < 0.05). Additionally, the *S.su* and *W.gl* groups demonstrated longer average swimming durations than the other groups, suggesting a potential trend toward improved welfare in betta fish. Aquatic plants shaped the microbial diversity and composition within the experimental aquatic system. The *W.gl* group had the highest microbial diversity, and the *A.re* and *S.su* groups enriched Verrucomicrobiota. These results demonstrate the preferential shaping of microbial communities by aquatic plants, suggesting a potential pathway for enhancing water quality. In conclusion, *S. subulata* demonstrates the greatest benefits under the experimental conditions, making it a more suitable choice for this experiment.

## Simple Summary

Maintaining stable conditions in small, unfiltered aquaria for betta fish is a significant challenge. This study investigated whether adding aquatic plants could serve as a natural biofilter and provide environmental enrichment to improve the aquatic environment and fish welfare. Over 25 days, aquaria containing aquatic plants, such as *Sagittaria subulata*, *Alternanthera reineckii*, and *Wolffia globosa*, compared to a plant-free control, were monitored. We found that the aquatic plants, particularly *S. subulata*, effectively reduced ammonia nitrogen. Furthermore, fish in aquaria planted with *S. subulata* displayed the longest average active swimming time and fewer negative behaviors. The plants also fostered a more diverse water microbial community at the end of the experiment. These results demonstrate that adding aquatic plants tends to improve the water quality and animal welfare in newly established betta fish tanks.

## 1. Introduction

**Figure 1:**
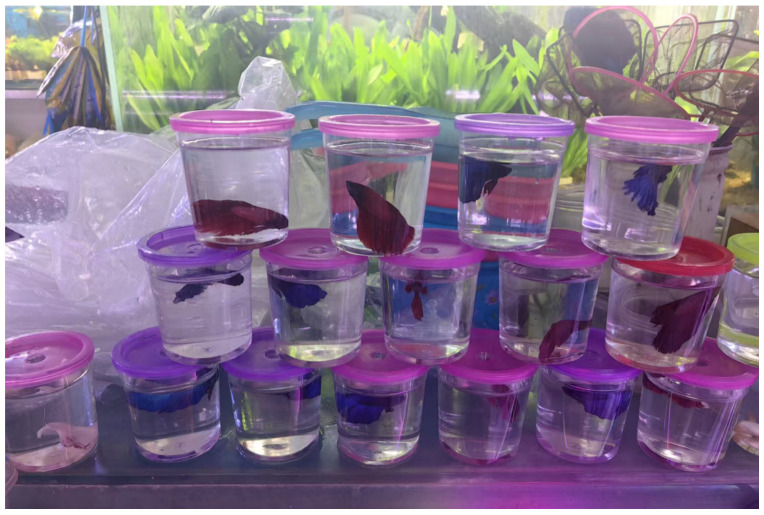
Housing conditions of betta fish in retail environments.

The betta fish (*Betta splendens*), commonly known as the Thai betta, is an ornamental fish species native to Southeast Asia and popular in the aquarium trade. Since 2019, it has gradually gained prominence in the market and has become a significant aquatic resource, driving regional economic development [1]. Characterized by conspicuous coloration and elongated finnage, it has become a commercially important species in the global ornamental fish trade and is now routinely maintained in both public aquaria and domestic collections. The unique appearance of betta fish has been extensively used across various art industries, which has not only enhanced their public profile but also generated millions of U.S. dollars in revenue for Thailand [1]. Owing to their pronounced territoriality and aggression, and facilitated by the presence of an accessory breathing organ known as the labyrinth organ, betta fish are often housed individually in small containers or rectangular aquaria, as shown in Figure 1.

The limited water volume in these small enclosures precludes the installation of aeration and filtration equipment, making it difficult to maintain an adequate water quality. Water quality deterioration is a critical risk in fish farming. An aquarium can be considered a static, semi-closed aquatic environment during the culture process. In such systems, the decomposition of fish feces, uneaten feed, and other organic matter generates both solid and dissolved pollutants. These pollutants lead to detrimental shifts in key water quality parameters, including dissolved oxygen (DO), ammonia nitrogen (NH_3_-N), and potential of hydrogen (pH).

Deteriorated water quality is a prevalent environmental stressor for fish, eliciting a tripartite stress response. The primary response involves neuroendocrine activation, characterized by the release of stress hormones, including cortisol and adrenaline. The secondary response is characterized by the dysregulation of energy metabolism, hematological parameters, biochemical processes, and immune function. The tertiary response ultimately compromises organismal performance (e.g., growth, reproduction, and disease resistance) and behavior, including abnormal swimming patterns [2,3,4].

Whether after short-term or long-term exposure to poor conditions, the risk of opportunistic and pathogenic infections, including immunosuppression, will increase, which can result in high mortality. Even survivors may subsequently die from secondary diseases or osmotic dysfunction [5]. For instance, a decline in DO often accompanies elevated carbon dioxide levels, resulting in respiratory acidosis and nephrocalcinosis, which significantly impairs growth [6,7]. Similarly, when the NH_3_-N exceeds 0.14 mg·L^−1^, it negatively affects fish performance and diminishes their disease resistance [8]. At even higher concentrations, it induces pathological alterations in the gill, kidney, and liver tissues of fish [6]. In brief, poor water quality severely compromises survival rates [5], reduces metabolic and ventilation rates, and triggers behavioral alterations [6]. Behavioral alterations in fish represent the most straightforward and valid measure of well-being and stress responses [9].

The microbial community is fundamental to ecosystem stability and fish well-being in aquaria. Microorganisms in the water column and sediments are critical for decomposing organic waste, maintaining chemical balance by processing nitrogen, and competing with potential pathogens on fish skin and mucus [10,11,12]. The stability of the microbial community is particularly important in small aquaria. The community is highly sensitive to environmental changes. Once the ecosystem is disrupted and microbial homeostasis is lost, the abundance of potential pathogens increases, sharply elevating the risk of disease outbreaks [13,14]. This stability is particularly challenged in newly established aquaria, where a functional nitrification system is insufficient. In the absence of beneficial bacteria, fish waste rapidly accumulates, creating a stressful and detrimental environment. For example, DO, pH, and NH_3_-N represent prevalent environmental determinants that significantly influence microbial community dynamics. Low DO levels not only reduce microbial diversity [15,16] but also result in black and odorous water [17]. Low DO levels also inhibit nitrifying bacteria, reducing the nitrogen removal efficiency [18]. Changes in pH inhibit nitrifying bacteria [19,20], leading to NH_3_-N accumulation. A decline in nitrifying bacteria efficiency can promote the proliferation of specific bacterial taxa, such as Proteobacteria, and potentially pathogenic species, including *Aeromonas*, thereby disrupting ecological equilibrium and posing a direct threat to fish health [11,21]. Furthermore, alterations in the aquatic microbial community may subsequently modulate the gut microbiota of cultured species, thereby influencing their immune competence and growth performance [22,23].

Consequently, the interaction between water environmental factors and the developing microbial community is a critical determinant of success for a new aquarium. To maintain stable water quality and mitigate disease outbreaks, multiple management strategies have been implemented in aquaculture. However, for small ornamental fish with limited space, such as betta fish, traditional physical and biological filtration systems are often difficult to apply. Aquatic plants constitute an integral and enduring component of natural aquatic ecosystems, serving a pivotal function in home aquaria through oxygen production, water quality enhancement, food provision, and the suppression of detrimental bacteria [24,25]. Moreover, aquatic plants contribute to regulating water quality by directly absorbing nutrients such as nitrates and indirectly controlling their buildup within the aquatic ecosystem [26]. Aquatic plants influence microbial communities by altering the water environment. Meanwhile, alterations in the structure, abundance, and function of the aquatic microbial community influence fish growth and swimming behaviors [27,28]. Aquatic plants also provide shelter for fish, reducing stress and improving their welfare [29].

To study the effects of aquatic plants on the water environment and fish welfare in newly established betta fish tanks, and considering that different aquatic plants have varying impacts on water quality, this experiment selected three popular, easy-to-cultivate aquatic plants suitable for betta tanks, including *Sagittaria subulata*, *Alternanthera reineckii*, and *Wolffia globose*, and added them, respectively. *S. subulata* is a common aquarium plant belonging to the genus *Sagittaria* of the Alismataceae family. This species features narrow, linear submerged leaves and broader, lanceolate emergent leaves. It also has a well-developed root system and grows rapidly, enabling it to thrive fully submerged. *A. reineckii*, a species in the genus *Alternanthera* of the Amaranthaceae family, is characterized by its lanceolate to ovate leaves that display a distinctive burgundy-red coloration. It exhibits relatively slow growth and requires moderate-to-high lighting conditions. *W. globosa*, a species in the genus *Wolffia* of the Araceae family, is a prevalent aquatic plant characterized by its small, elliptical green fronds. It exhibits a rapid growth, high reproductive capacity, strong environmental adaptability, and substantial nutritional value. In this experiment, we selected three morphologically distinct aquatic plants to harness their ecological functions—NH_3_-N removal by roots, oxygen release via photosynthesis, and microbial community restructuring—thereby improving the aquatic environment for betta fish. The subsequent analysis of water quality parameters, microbial composition, and fish behavior over the experiment offers evidence-based insights for betta husbandry.

## 2. Materials and Methods

### 2.1. Ethical Approval

Our experiments were approved by the science and technology ethics committee of Northeast Forestry University (# 2025-125).

### 2.2. Experimental Materials and Culture Conditions

Twelve betta fish of the same batch used in the experiment were purchased from the Harbin Dao Wai Aquarium Market, with body lengths of 5.53 ± 0.51 cm. *S. subulata*, *A. reineckii*, and *W. globosa* were also purchased from the aquarium market. After purchasing, all plants were immediately soaked in 5 ppm potassium permanganate for 15 min, then rinsed with clean water before being placed in the aquarium.

Each betta fish was individually housed in one glass aquarium (12 × 12 × 15 cm) filled with 2 L of well-aerated water. No filtration or aeration devices were installed in any tank. To minimize external disturbance and the impact on betta fish, three sides of each aquarium were covered with white paper, leaving only one side for observation. A white mesh cloth was placed over the top to prevent jumping. The rearing water was pre-aerated tap water, maintained at 21 ± 2 °C. A 1.2 m LED lamp (Foshan Lighting, Foshan, China) was installed as the illumination source. The light source provided a spectral range of 400–700 nm, and the illuminance at the experimental area was maintained at approximately 2000–4000 lux. Additionally, the experimental photoperiod was set at a 14:10 h light–dark (L:D) cycle. Finally, fish were manually fed a small amount of high-protein, betta-specific diet daily at 07:00 and 17:00. The ration was adjusted to the amount consumed within two minutes. After feeding, the feces and leftover food was immediately sucked up, and the dead or withered leaves of the plants were trimmed. A small amount of well-aerated water was added daily to maintain a constant water level.

### 2.3. Experimental Design

**Figure 2:**
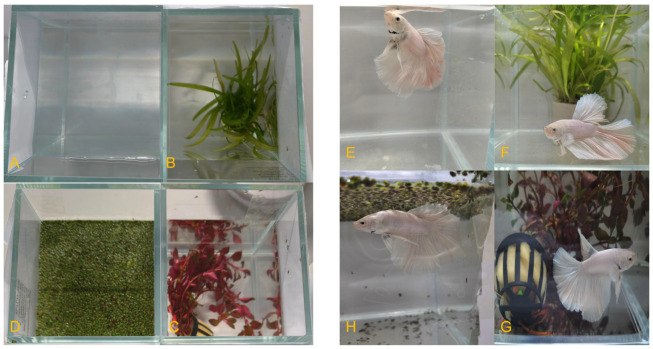
Experimental setup of betta aquaria. Note: (**A**–**D**) represent the top view for the CG group, *S.su* group, *A.re* group, and *W.gl* group, respectively. (**E**–**H**) represent the side view for the CG group, *S.su* group, *A.re* group, and *W.gl* group, respectively.

Four groups were set up, including an *A. reineckii* (*A.re*) group, *S. subulata* (*S.su*) group, *W. globosa* (*W.gl*) group, and control (CG) group with no plants, with three replicates per group in a 25-day trial. The plant biomass added to each tank was 8 g in three treatment groups. Each group was formulated as indicated in Figure 2. Water parameters (DO and pH) were measured on days 1–25 d, and NH_3_-N was measured on days 1, 2, 3, 4, 5, 6, 7, 10, 13, 16, 19, 22, and 25; behavioral analyses were conducted on days 1, 2, 3, 4, 5, 6, 9, 12, 15, 18, 21, and 24. Microbial community analysis was performed using water samples collected at the end of the experiment. After water sampling was completed, all aquatic plants were removed from the tanks, rinsed, gently blotted dry, and weighed to obtain their fresh weight for the subsequent calculation of the growth rate in each group. Plant biomass growth was assessed using the fresh weight method. While straightforward, this approach may be influenced by variations in the plant tissue water content. Daily maintenance included waste removal with sterile pipettes after feeding, followed by water replenishment to 2 L. During the experiment, the total amount of water replaced was about one-fourth of the total volume, similar to the routine maintenance of a home aquarium.

### 2.4. Behavioral Observation

Based on behavioral ethograms from Oldfield et al. [30,31,32] and our preliminary observations, several classic behavioral patterns of betta fish were selected for analysis. The behavior ethograms were as follows (Table 1). A 5 min segment was randomly extracted from each recorded video for behavioral analysis.

### 2.5. Water Quality Measurement and Microbial Community Sample Collection

DO and pH were measured daily at 17:00 using the JPB-607A portable DO meter (Leici, Shanghai, China) and the pen-type pH meter (SAMRT sensor, Dongguan, China), respectively. NH_3_-N was determined once daily at 17:00 using Nessler’s method [33].

On the final day of the experiment, 100 mL of uniformly mixed water was collected from each aquarium. The samples were sequentially vacuum-filtered through 0.45 and 0.22 μm membrane filters (Xinya, Shanghai, China), and each filter was then transferred into a 15 mL sterile centrifuge tube (NEST, Wuxi, China). All samples were immediately flash-frozen in liquid nitrogen and stored at −80 °C for subsequent analysis. It should be noted that microbial community sampling was conducted only at the endpoint of this study, therefore precluding the observation of its dynamic changes.

### 2.6. DNA Extraction

Total community genomic DNA extraction was performed using an E.Z.N.A™ Mag-Bind Soil DNA Kit (Omega, M5635-02, Norcross, GA, USA), following the manufacturer’s instructions. We measured the concentration of the DNA using a Qubit 4.0 (Thermo, Waltham, MA, USA) to ensure that adequate amounts of high-quality genomic DNA had been extracted.

### 2.7. 16S rRNA Gene Amplification by PCR

Our target was the V3–V4 hypervariable region of the bacterial 16S rRNA gene. PCR was started immediately after the DNA was extracted. The 16S rRNA V3–V4 amplicon was amplified using 2× Hieff^®^ Robust PCR Master Mix (Yeasen, 10105ES03, Shanghai, China). Two universal bacterial 16S rRNA gene amplicon PCR primers (PAGE purified) were used: the amplicon PCR forward primer (CCTACGGGNGGCWGCAG) and amplicon PCR reverse primer (GACTACHVGGGTATCTAATCC). The reaction was set up as follows: microbial DNA (10 ng/µL), 2 µL; amplicon PCR forward primer (10 µM), 1 µL; amplicon PCR reverse primer (10 µM), 1 µL; 2× Hieff^®^ Robust PCR Master Mix (Yeasen, 10105ES03, China) (total 30 µL). The plate was sealed, and PCR was performed in a thermal instrument (Applied Biosystems 9700, Foster City, CA, USA) using the following program: 1 cycle of denaturing at 95 °C for 3 min, first 5 cycles of denaturing at 95 °C for 30 s, annealing at 45 °C for 30 s, elongation at 72 °C for 30 s, then 20 cycles of denaturing at 95 °C for 30 s, annealing at 55 °C for 30 s, elongation at 72 °C for 30 s, and a final extension at 72 °C for 5 min. The PCR products were checked using electrophoresis in 2% (*w*/*v*) agarose gels in TBE buffer (Tris, boric acid, EDTA) stained with ethidium bromide (EB) and visualized under UV light.

### 2.8. 16S Gene Library Construction, Quantification, and Sequencing

We used Hieff NGS™ DNA Selection Beads (Yeasen, 10105ES03, China) to purify the free primers and primer dimer species in the amplicon product. Samples were delivered to Sangon BioTech (Shanghai, China) for library construction using the universal Illumina adaptor and index. Before sequencing, the DNA concentration of each PCR product was determined using a Qubit^®^ 4.0 Green double-stranded DNA assay, and it was quality controlled using a bioanalyzer (Agilent 2100, Santa Clara, CA, USA). Depending on the coverage needs, all libraries can be pooled for one run. The amplicons from each reaction mixture were pooled in equimolar ratios based on their concentration. Sequencing was performed using the Illumina MiSeq system (Illumina MiSeq, San Diego, CA, USA), according to the manufacturer’s instructions.

### 2.9. Sequence Processing, OTU Clustering, Representative Tags Alignment, and Biological Classification

After sequencing, the two short Illumina readings were assembled using PEAR software (version 0.9.8) according to the overlap, and fastq files were processed to generate individual fasta and qual files, which could then be analyzed through standard methods. The effective tags were clustered into operational taxonomic units (OTUs) of ≥97% similarity using Usearch software (version 11.0.667). Chimeric sequences and singleton OTUs (with only one read) were removed, after which the remaining sequences were sorted into each sample based on the OTUs. The tag sequence with the highest abundance was selected as a representative sequence within each cluster. Bacterial and fungal OTU representative sequences were classified taxonomically by blasting against the RDP Database and UNITE fungal ITS Database, respectively. To profile the microbial community structure, the number of sequences assigned to each taxon was counted for every sample at different taxonomic levels (phylum, class, order, family, genus, and species). The composition was then expressed as a relative abundance for subsequent comparative analysis.

### 2.10. Statistical Analysis

Data were processed in Microsoft Excel (version 2021), and a scatter plot was generated in Origin (version 2024). Data were analyzed statistically with SPSS (version 23.0), employing one-way analysis of variance (ANOVA) followed by LSD multiple comparison tests to evaluate the effects of different aquatic plants on the α-diversity of microbial communities and the growth rate of aquatic plants. Linear mixed-effects models (LMMs) were fitted for the response variables, including dissolved oxygen (DO), pH, ammonia nitrogen (NH_3_-N), and fish behaviors. Treatment and day were included as fixed effects, while tank ID (1–12) was incorporated as a random effect to account for repeated measures. When a significant main effect of treatment was detected (*p* < 0.05), post hoc pairwise comparisons were conducted using the Bonferroni correction. Results are expressed as means ± SE, with statistical significance defined at *p* < 0.05.

The α-diversity indices (including Ace, Chao1, Simpson, and Shannon indices) were quantified in terms of OTU richness. To assess sample adequacy, rarefaction curves of the observed numbers of OTUs were constructed, and all α diversity indices were calculated with Mothur software (version 3.8.31). The OTU rarefaction curve was plotted in R (version 3.6.0). To estimate the diversity of the microbial community of the sample, we calculated the within-sample (alpha) diversity using the ANOVA test for multiple group comparisons. Beta diversity evaluates differences in the microbiome among samples. We performed constrained principal coordinate analysis (PCoA) using the vegan package (version 2.5–6) in R software. Stacked bar plots showing dominant species relative abundance were created using R software. To further analyze environment–sample relationships, redundancy analysis (RDA) plots were generated with the R vegan package. Additionally, heatmaps of species–environment factor correlations were drawn using the R corrplot package (version 0.84) to assess microbe–environment linkages.

## 3. Results

### 3.1. Effects of Aquatic Plants on Water Quality in Newly Established Betta Aquaria

**Figure 3:**
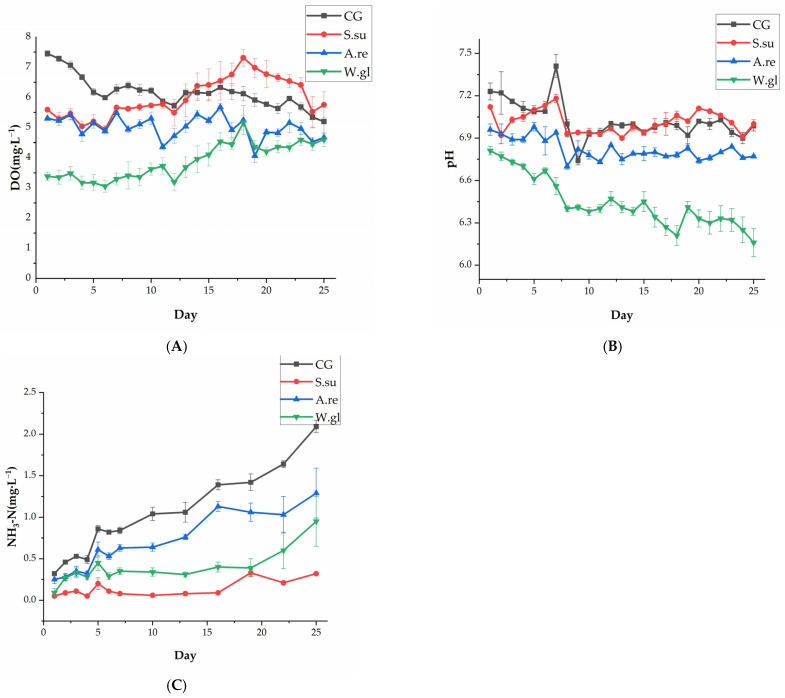
Dynamics of DO (**A**), pH (**B**), and NH_3_-N (**C**) in betta fish tanks.

The trend of DO changes in all groups during the experimental period is shown in Figure 3A. The CG group initially showed the highest DO during days 1–13, which was gradually surpassed by the *S.su* group after day 14. The *S.su* group reached its highest value of 7.31 mg·L^−1^ on day 18. The DO in the *A.re* group fluctuated around 5 mg·L^−1^, while the *W.gl* group consistently maintained the lowest levels, despite a slight increase over time. The mean values of the DO parameters are described in Table S1.

The trend of pH changes in all groups during the experimental period is shown in Figure 3B. The pH values remained relatively stable in the CG and *S.su* groups, ranging between 7.0 and 7.2. In contrast, the *A.re* group stabilized at a slightly lower level after a minor initial decline. The *W.gl* group exhibited the most pronounced decrease in pH, consistently recording the lowest values among all groups, reaching a minimum value of 6.16 on day 25. The mean values of the pH parameters are described in Table S2.

The trends of NH_3_-N changes in all groups during the experimental period are shown in Figure 3C. NH_3_-N varied significantly in the four groups. The CG group demonstrated a marked and continuous increase, reaching the highest concentration of 2.09 mg·L^−1^ on day 25. The *A.re* group also showed a clear upward trend from day 5, exceeding both the *W.gl* and *S.su* groups. The *W.gl* group’s NH_3_-N fluctuated around 0.3 mg·L^−1^ until day 19 before rising sharply. Notably, the *S.su* group maintained the lowest and most stable NH_3_-N levels throughout the study. The mean values of the NH_3_-N parameters are described in Table S3.

The linear mixed model analysis showed that both aquatic plants and time had a significant effect on the DO, pH, and NH_3_-N in the betta fish tank water (*p* < 0.05). The DO, pH, and NH_3_-N levels in the *A.re*, CG, and *S.su* groups all showed significant differences compared to the *W.gl* group (*p* < 0.05).

Bonferroni post hoc tests revealed significant differences in DO among the different groups. Table 2 shows that, by comparing the water quality parameters of each group over the 25-day experimental period, it was found that the DO and pH of the CG group and *S.su* group were significantly higher overall than those of the *A.re* group and *W.gl* group (*p* < 0.05). The DO level and pH value of the *A.re* group were significantly higher overall than those of the *W.gl* group (*p* < 0.05). The NH_3_-N content of the CG group was significantly higher overall than that of the other three groups (*p* < 0.05). The NH_3_-N content of the *A.re* group was significantly higher overall than that of the *W.gl* and *S.su* groups (*p* < 0.05). The NH_3_-N content of the *W.gl* group was significantly higher overall than that of the *S.su* group (*p* < 0.05), and the NH_3_-N content of the *S.su* group was significantly lower overall than that of the other groups (*p* < 0.05).

### 3.2. Effects of Different Aquatic Plants on Betta Fish Behavior

**Figure 4:**
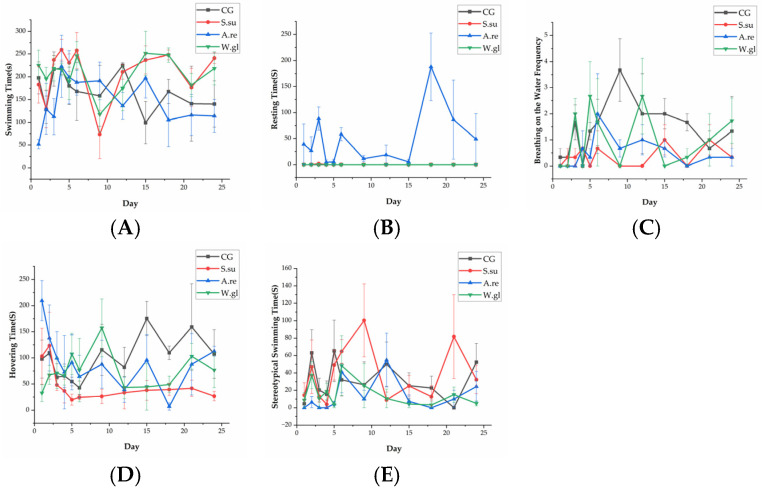
The changes in behavior of betta fish of swimming (**A**), resting (**B**), breathing on the water (**C**), hovering (**D**), and stereotypical swimming (**E**), respectively.

Figure 4 depicts the behavioral profiles of betta fish in all groups. Swimming duration varied considerably, with the *S.su* and *W.gl* groups showing relatively prolonged activity during days 3–6 and 15–21, respectively, whereas the *A.re* group exhibited frequent reductions on 1–4 d, 12 d, and 18–24 d. Notably, resting behavior was nearly exclusive to the *A.re* group throughout the trial period based on the video recordings. The surface breathing frequency was highest in the CG, particularly on days 9 and 15–24, and *W.gl* groups on days 2, 5–6, 12, and 24, but minimal in the *S.su* group. On days 1–4, the *A.re* group mostly hovered. On days 5–9, hovering was most common in the W.gl group. The CG showed the most hovering from days 12–24. After day 3, the *S.su* group showed a consistently low hovering time. Stereotypical swimming was most pronounced in the CG on days 2, 5, and 24 and in the *S.su* group during days 6–9 and 21–24. The mean value of the behaviors is described in Tables S4–S8.

The linear mixed model analysis indicated that aquatic plants had a significant effect on betta fish behavior (*p* < 0.05). Table 3 compares the behaviors of bettas in each group over a 25-day experimental period. The analysis shows that there was no significant difference in the swimming, hovering, or stereotypical swimming time of bettas among all groups. Bettas in the *A.re* group exhibited resting behavior significantly more than the other three groups (*p* < 0.05). The frequency of surface breathing behavior in the CG group was significantly higher than in the *S.su* and *A.re* groups (*p* < 0.05).

### 3.3. Temporal Changes in Plant Biomass and Growth

Table 4 presents the final weight and growth rate of aquatic plants in each group at the end of the experiment. The *S.su* group showed a significantly higher weight gain compared to the other groups (*p* < 0.05), whereas the *W.gl* group exhibited a clear negative growth rate.

### 3.4. Effects of Different Aquatic Plants on Microbial Community Structure

#### 3.4.1. Sequencing Data Quality Assessment

**Figure 5:**
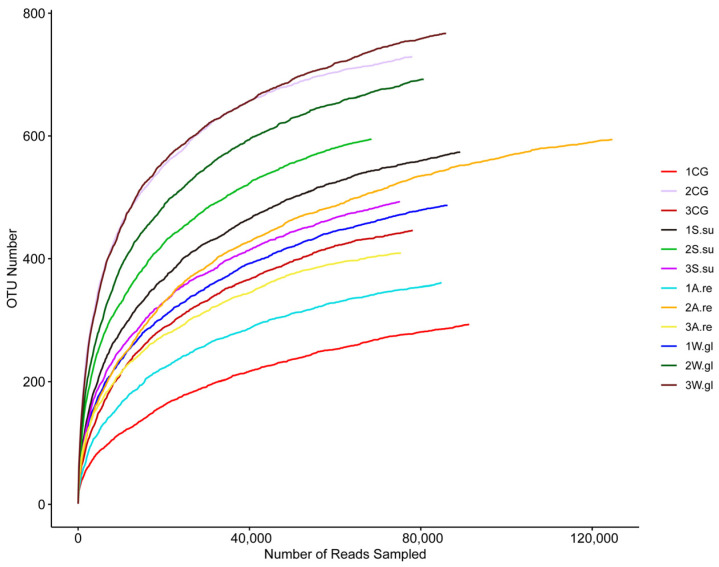
Rarefaction curves of alpha diversity indices.

16S rRNA sequencing of the 12 water samples yielded 1,278,140 high-quality sequences, with an average length of 416.90 base pairs (bp). A cluster analysis at 97% sequence similarity identified 1409 operational taxonomic units (OTUs). Representative OTU sequences were compared against reference databases for taxonomic annotation, resulting in the identification of 28 phyla, 58 classes, 149 orders, 245 families, 459 genera, and 663 species. The OTU rarefaction curve (Figure 5) reached a clear plateau with increasing sequencing depth, indicating an adequate sequence coverage for reliable downstream bioinformatic analysis and a faithful representation of the microbial composition.

#### 3.4.2. Microbial Alpha Diversity

Alpha diversity metrics were used to assess microbial community characteristics. The Chao1 and Ace indices estimate the total number of OTUs, reflecting species richness. The Shannon and Simpson indices describe species diversity: a higher Shannon value indicates greater diversity, while a higher Simpson value denotes lower diversity.

In this experiment, the Shannon, Simpson, Ace, and Chao1 indices collectively demonstrated that adding different aquatic plants influenced the microbial diversity in betta aquaria (Table 5). Specifically, the *W.gl* group exhibited a significantly higher Ace index than the CG group. The Chao1 index in the *W.gl* group was significantly greater than that of both the CG and *A.re* groups. Furthermore, the *W.gl* group showed a significantly higher Shannon index compared to the other three groups, while its Simpson index was significantly lower than that of the *A.re* group. These results show that the *W.gl* group had the richest microbial community. Its microbial richness was significantly higher than all other treatment groups.

#### 3.4.3. Analysis of Microbial Beta Diversity

**Figure 6:**
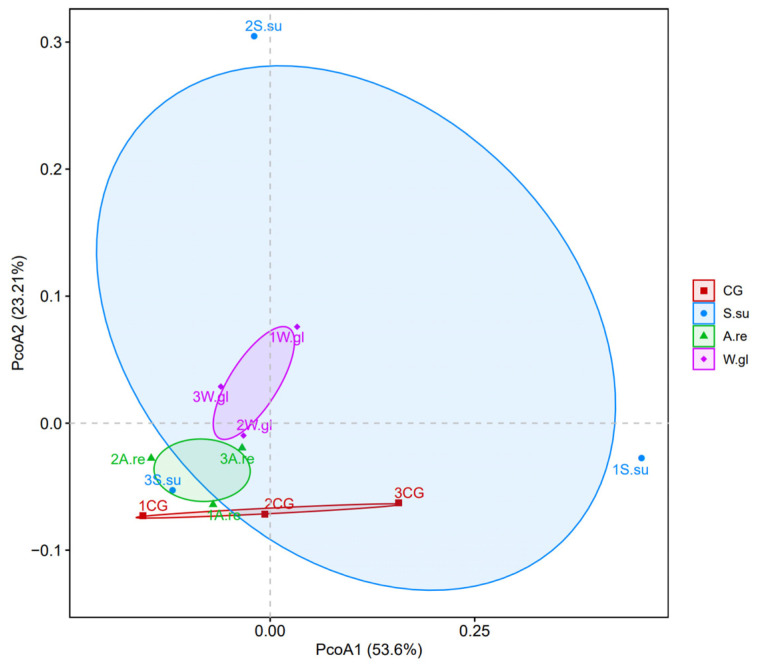
Principal coordinate analysis (PCoA) plot.

Principal coordinate analysis (P CoA) was conducted to assess differences in the microbial community composition among groups (Figure 6). In the P CoA plot, samples from different treatment groups are represented by distinct colors or shapes. The proximity between any two samples reflects their degree of similarity in species composition. A comparative analysis of the microbial community structure at the phylum level revealed that the first principal coordinate (PCoA1) accounted for 53.60% of the total variance, while the second (PCoA2) explained 23.21%, resulting in a cumulative explanatory power of 76.81%. Samples from the *W.gl* group formed a tight cluster, indicating a high similarity and low variability in the microbial community structure within this group. Similarly, samples in the *A.re* group were positioned close to one another, suggesting a relatively stable community composition within this treatment. In contrast, samples from the CG group showed a greater dispersion compared to the planted groups, reflecting distinct microbial community characteristics. The *S.su* group samples were clearly separated from the other three groups, demonstrating substantial differences in the microbial community structure both within the group and compared to other treatments.

#### 3.4.4. Composition of Aquatic Microbial Community

**Figure 7:**
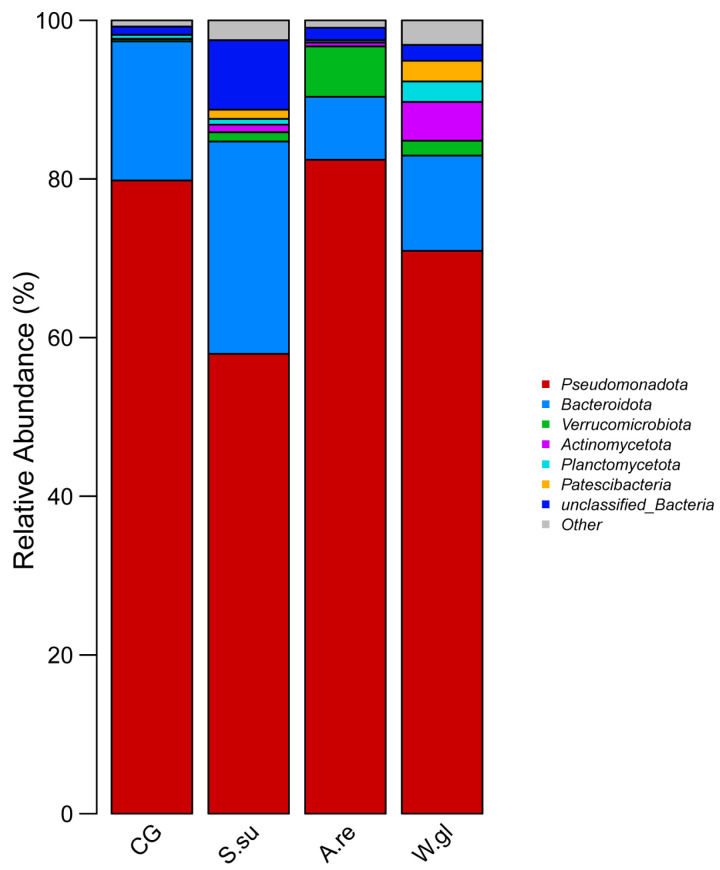
Stacked bar plot of relative abundance for dominant taxa at the phylum level.

An analysis at the phylum level revealed distinct microbial community profiles among the different groups. The aquatic microbial communities in the betta aquaria were primarily composed of Pseudomonadota, Bacteroidota, Verrucomicrobiota, and Actinomycetota (Figure 7). For all groups, Pseudomonadota and Bacteroidota were the dominant phyla. However, adding aquatic plants, particularly *A.reineckii* and *W.globosa*, substantially reshaped the microbial community structure in newly established betta aquaria. For example, Verrucomicrobiota was the third most dominant phylum in both the *A.re* and *S.su* groups, Actinomycetota prevailed in the *W.gl* group, while Planctomycetota ranked third in the CG group at a minimal relative abundance of 0.51%.

**Figure 8:**
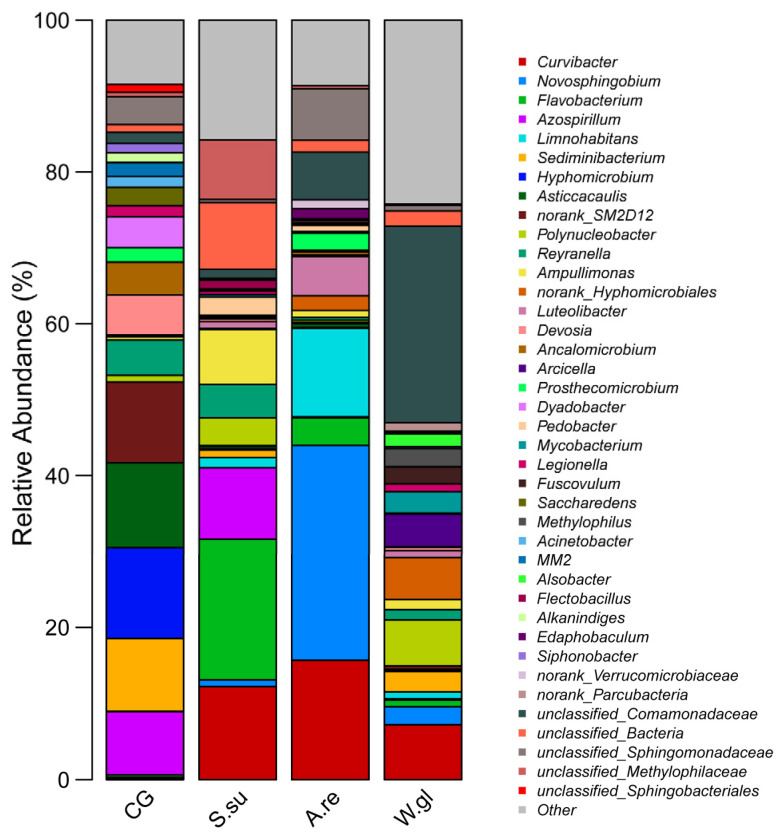
Stacked bar plot of relative abundance for dominant taxa at the genus level.

Figure 8 reveals distinct microbial compositions at the genus level across the four groups. The nitrogen-fixing bacteria *Azospirillum* maintained a high abundance in both the CG group and the *S.su* group, while *Sediminibacterium* and *Hyphomicrobium*, which participate in the nitrification process, dominated in the CG group. In terms of organic matter degradation function, various plant groups generally enriched bacterial genera with related capabilities, such as *Flavobacterium* in the *S.su* group, *Novosphingobium* and *Limnohabitans* in the *A.re* group, and *Curvibacter*, commonly found in all plant groups. It is worth noting that *Asticcacaulis* enriched in the CG group and *Mycobacterium* appearing in the *W.gl* group are both potentially risky bacterial groups. In addition, *Polynucleobacter*, which is unique to the *W.gl* group, and *Luteolibacter* in the *A.re* group are often regarded as indicator bacteria for good water quality.

Notably, the *Azospirillum* was a dominant genus shared by the CG and *S.su* groups. *Curvibacter* was common to all three planted groups. Taxonomically, the most dominant genera (e.g., *Hyphomicrobium*, *Azospirillum*, *Curvibacter*, *Novosphingobium*) belong to the phylum Pseudomonadota. Others were affiliated with Bacteroidota (e.g., *Sediminibacterium*, *Flavobacterium*), Verrucomicrobiota (e.g., *Luteolibacter*), and Actinomycetota (e.g., *Mycobacterium*).

#### 3.4.5. Relationship Between Microbial Community Structure and Environmental Factors

**Figure 9:**
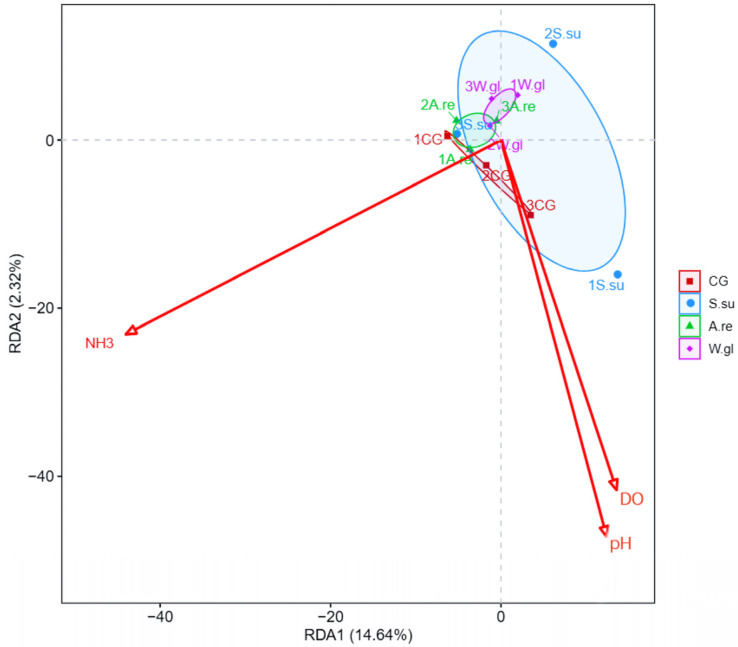
Redundancy analysis (RDA).

A redundancy analysis (RDA) was employed to investigate the interactions between the microbial community structure and key environmental parameters. The RDA revealed that the first two axes (RDA1 and RDA2) explained 14.64% and 2.32% of the total variance, respectively, with a cumulative explanation rate of 16.96% (Figure 9). The microbial community structure in the CG group was most strongly influenced by environmental factors. The degree of influence of environmental factors on the microbial community of *A.re* and *W.gl* groups was not significantly different, while the influence on the *S.su* group was the least.

**Figure 10:**
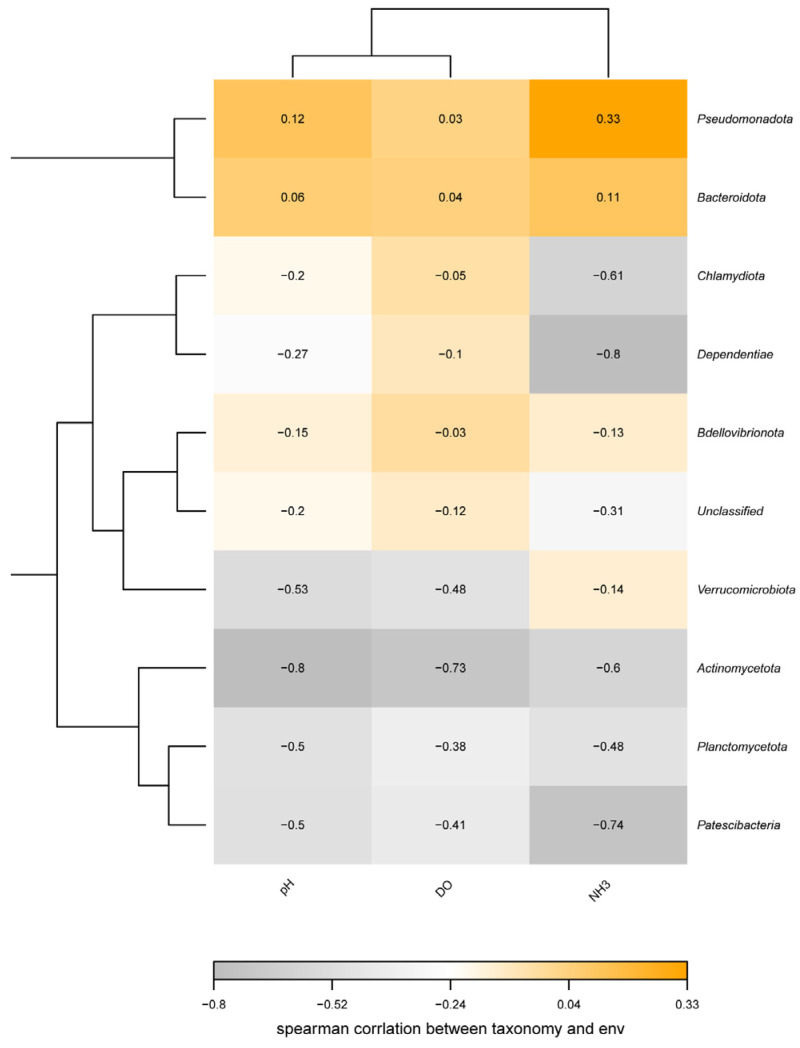
Correlation heatmap between environmental factors and dominant bacterial phyla.

A correlation analysis (Figure 10) indicated that the relative abundances of Pseudomonadota and Bacteroidota were positively correlated with NH_3_-N, DO, and pH levels. In contrast, Actinomycetota, Verrucomicrobiota, and Planctomycetota showed negative correlations with these same environmental factors.

## 4. Discussion

### 4.1. Effects of Aquatic Plants on Water Quality in Betta Aquaria

#### 4.1.1. DO

DO is a critical environmental factor in aquaculture. Hypoxia serves as a physiological stressor, impairing immune function and elevating disease susceptibility [34], which negatively impacts survival and growth. DO levels are affected by the rates of plant photosynthesis, respiration from fish, plants, and microorganisms, and air–water gas transfer [35]. Water quality often declines due to poor management, while fish respiration and bacterial decomposition of residual feed and feces consume oxygen, leading to a decreased DO. As a result, the CG group showed a declining trend in DO during the construction of a new betta fish tank. In our study, we observed significant differences in DO levels under various aquatic plant treatments. These differences may be directly or indirectly related to the distinct morphological traits and growth stages of the plants. Aquatic plants release oxygen produced through photosynthesis via their aerenchyma tissues into the root zone, subsequently increasing DO in the surrounding water through radial oxygen loss [36]. The dense green foliage, well-developed root system, submersible habit, and rapid growth of *S. subulata* likely caused the high DO during the mid-to-late stages of the experiment. Previous research has shown that planting *Elodea canadensis* significantly raises water DO levels through photosynthesis [37]. Conversely, *A. reineckii* has thicker red leaves that poorly absorb white light and show growth stagnation throughout the trial. This indicates its limited contribution to photosynthetic oxygen production, consistent with the low DO levels observed in the *A.re* group. The dense surface cover of *W. globosa* may hinder atmospheric oxygen exchange and shade the water column. This reduction in light availability likely suppresses photosynthetic oxygen production from both the plants themselves and phytoplankton, leading to lower DO [38]. Adequate oxygen helps speed up nitrification and denitrification, promoting plant growth and creating a positive feedback loop that further raises DO levels [39]. The notable decline in *W. globosa* biomass at the end of the experiment probably indicates tissue decay, which promotes heterotrophic microbial decomposition, leading to oxygen consumption and explaining why this group had the lowest DO levels. Introducing aquatic plants into new aquaria involves a complex adaptation process, including the reorganization and regulation of photosynthetic activity. Therefore, the lack of high DO levels from *S. subulata* in the early stages may be due to its inability to adapt quickly to the new aquatic environment [40]. In summary, under the experimental conditions, adding *S. subulata* seems to better promote plant growth and photosynthesis, helping to maintain stable DO levels in betta aquaria.

#### 4.1.2. pH

In aquatic systems, deviations from optimal pH can severely compromise metabolism. A high pH promotes toxic NH_3_-N accumulation, whereas an excessively low pH reduces the oxygen-carrying capacity of organisms [41]. Photosynthesis by aquatic plants releases oxygen while consuming dissolved carbon dioxide, consequently elevating the water pH [42]. Previous studies have documented that *Oenanthe javanica* and *Thalia dealbata* resulted in an initial pH increase, followed by a stabilization over one month [43]. In our study, all groups exhibited a general decline in pH over the experimental period, contrary to the expected diurnal patterns. This result suggests that, in small, closed aquatic systems, acidification may be the dominant factor shaping water chemistry in betta fish culture.

The observed pH decline in planted aquaria over 25 days may be attributed to a combination of factors, including plant-mediated pollutant purification in a small water volume [44], hydrogen ion release associated with macrophyte ammonium uptake [45], and hydrogen ion production from bacterial nitrification [46]. Furthermore, betta fish respiration contributed carbon dioxide, further acidifying the water. However, our study revealed a diverging effect of different aquatic plants on water pH. Most notably, the *W.gl* group exhibited a consistent and significant decreasing trend. We hypothesize that this is attributable to the extensive surface coverage by *W. globosa*, which likely restricted atmospheric gas exchange and light penetration, thereby limiting the complete consumption of dissolved CO_2_. Furthermore, the significant biomass reduction in the *W.gl* group suggests frequent occurrences of plant decay and decomposition within the aquarium. Both heterotrophic microbial decomposition and the breakdown of plant cells release acidic substances into the water, which likely contributed to the persistent pH decline [47], potentially in conjunction with the aforementioned surface coverage effect. Interestingly, *S. subulata* also maintained a relatively stable pH trend. This stability appears to be closely linked to the concurrently higher DO levels. We propose that the sustained oxygen production by *S. subulata* may have offset the CO_2_ and acids generated by microbial and fish respiration, thus contributing to pH homeostasis.

#### 4.1.3. NH_3_-N

NH_3_-N is a common aquatic pollutant derived from the decomposition of organic matter and exerts toxic effects on aquatic animals [48]. Its removal in water primarily occurs through plant uptake, nitrification, and ammonia volatilization [49]. Aquatic plants are recognized as ecologically significant plant species capable of taking up environmental pollutants from their surroundings [50]. Aquatic plants absorb nitrogen through both root systems and photosynthetic tissues, assimilating it into structural biomass and consequently slowing nutrient cycling in aquatic environments [51]. The cultivation of aquatic plants creates more diverse habitats for microorganisms, thereby enhancing pollutant degradation [52].

In our study, NH_3_-N increased over the experimental period in all groups. The CG group exhibited the highest NH_3_-N concentrations, whereas the three planted groups showed a relatively slower rate of increase in aqueous NH_3_-N, consistent with prior research. In contrast to the DO and pH results, the NH_3_-N concentration in the *A.re* group was significantly lower than that in the CG group. Furthermore, the *W.gl* group was substantially lower than the *A.re* group, and the *S.su* group, in turn, was considerably lower than the *W.gl* group. This pattern clearly demonstrates the differential capacity of these plants to absorb and utilize NH_3_-N. Floating-leaved plants demonstrate a superior efficiency in NH_3_-N removal compared to emergent species [52]. Zhang et al. [53] demonstrated that *Sagittaria sagittifolia* exhibits a significantly greater efficacy in purifying aquaculture pond wastewater than *Eichhornia crassipes* and *Alternanthera philoxeroides*. The residual biomass results are consistent with the notion that the multi-rooted, submersible, and fast-growing *S. subulata* effectively utilized organic matter in the water, which collectively enhanced its capacity to mitigate the rapid accumulation of NH_3_-N.

In sharp contrast, the residual biomass results from the *A.re* and *W.gl* groups indicated that *A. reineckii* showed no discernible growth over the 25 days, whereas *W. globosa* entered a state of decline. The poor NH_3_-N absorption observed in the *A.re* and *W.gl* groups is likely attributable to their growth state. This suggests that the environmental conditions provided in this experiment may not have been conducive to their growth or their efficacy in NH_3_-N removal. Additionally, NH_3_-N removal relies not only on uptake by aquatic plants but also on nitrification by microorganisms, most of which are aerobic. Therefore, the high DO levels in the *S.su* group likely provided an optimal habitat for these bacteria, enhancing the overall removal process.

### 4.2. Effects of Aquatic Plants on Fish Behavior

Fish behavior serves as a visual indicator of welfare, reflecting their responses to environmental changes and stressors [54]. Deteriorating water quality, particularly low DO, critically impacts aquaculture by triggering surface breathing in fish. This maladaptive behavior restricts normal respiration, induces oxidative stress, and ultimately compromises growth, reproduction, and survival [55]. Confinement-related factors, including limited space, also frequently restrict natural behaviors, often leading to hovering and stereotypical swimming under suboptimal conditions [30]. A study on betta fish welfare found that individuals in larger, enriched aquaria swam longer and rested less than those in smaller, barren tanks [56]. Therefore, “Resting” is classified as a negative behavioral indicator in this species [57]. Furthermore, behavioral alterations are often linked to disease states; for instance, bettas infected with common pathogens exhibit reduced activity levels [58,59].

Adding plants to aquarium systems provides multiple benefits, including the reduction of NH_3_-N and the elevation of dissolved oxygen levels. Fish maintained in environments with higher DO typically exhibit greater activity and improved health [24]. Certain aquatic plant species release antimicrobial compounds that inhibit the growth of pathogenic bacteria [60]. Additionally, the root systems of these plants offer habitats for microbial communities, promoting the proliferation of beneficial microorganisms that indirectly suppress pathogens [61], thereby enhancing fish well-being. Furthermore, aquatic plants serve as environmental enrichment within aquaria, providing shelter for betta fish and reducing stress responses. Studies have shown that more enriched environments lead to increased swimming behavior in bettas [30], whereas reduced enrichment often results in stereotypical behaviors [62]. In our study, the *S.su* and *W.gl* groups displayed more swimming activity, while the *S.su* group showed reduced surface breathing and stationary floating. These findings indicate that introducing suitable aquatic plants may help establish a low-stress environment for betta fish by maintaining higher DO and lower NH_3_-N levels in the water, which in turn could mitigate their stress-associated behaviors. Environmental coloration constitutes a significant factor influencing fish welfare. The increased frequency of negative behaviors observed in the *A.re* group may be associated with its distinctive reddish hue. Studies have documented that Nile tilapia (*Oreochromis niloticus*) exhibit avoidance responses toward red environmental enrichment [63]. A prolonged exposure to red-spectrum illumination can induce physiological stress and discomfort, ultimately impairing growth performance in fish [64,65]. Due to the short duration and fixed evening recording schedule, the incomplete behavioral analysis may limit the interpretability of the results.

### 4.3. Effects of Aquatic Plants on Microbial Community Structure and Diversity

The aquatic microbial community maintains a stable and balanced environment where microorganisms from both fish and water bodies work together to prevent colonization by exogenous pathogens. Microbial communities associated with aquatic plant roots may contribute to improving aquatic environments [66]. Considerable differences exist in the microbial communities associated with different types of aquatic plants, with a higher microbial diversity generally conferring a greater resistance to environmental fluctuations and helping to maintain ecosystem functions [67,68]. In our study, the microbial diversity in planted aquaria was higher than in the CG group, with the *W.gl* group showing the highest diversity. However, through water quality monitoring, we found that the DO of the *W.gl* group was the lowest, which is inconsistent with the results reported by Zhao et al. [15,16]. The opposite results may be due to low oxygen levels limiting the excessive proliferation of certain dominant bacterial communities, or due to the organic substances secreted by *W. globosa* promoting the growth of various bacterial communities. Our results indicate that adding aquatic plants, particularly *W. globosa*, to new betta aquaria tends to increase microbial diversity, thereby enhancing aquatic stability and creating more favorable conditions for betta survival.

Freshwater aquaculture systems typically harbor microbial communities predominantly composed of Actinobacteria, Verrucomicrobiota, Planctomycetota, Cyanobacteria, Patescibacteria, Pseudomonadota, and Bacteroidota [69]. In this study, the dominant phyla in betta aquaria were identified as Pseudomonadota, Bacteroidota, Verrucomicrobiota, and Actinomycetota, which aligns with the findings from Gilbert, Xiong et al. [70,71,72].

### 4.4. Differences in Dominant Genera Among Groups Within the Pseudomonadota Phylum

Pseudomonadota represents a redefined taxonomic group formerly classified within Proteobacteria, excluding the classes Epsilonproteobacteria, Deltaproteobacteria, and Oligoflexia. Pseudomonadota was the most dominant bacterial phylum across all treatment groups. This phylum comprises numerous Gram-negative bacteria that perform critical ecological functions, such as degrading organic pollutants, improving water quality, and inhibiting pathogens. Collectively, these activities reduce the accumulation of ammonia and nitrite, thereby creating favorable conditions for fish [73]. Pseudomonadaceae is particularly abundant in aquaculture environments, which promotes growth in crustaceans [74]. However, certain pseudomonads may cause fish diseases or stress responses, with some species producing metabolites that lead to gill cell lysis [75,76].

#### 4.4.1. Genera Related to the Nitrogen Cycle

In the CG group, which had the highest NH_3_-N concentration, we detected a high abundance of *Hyphomicrobium*, which participates in nitrogen and phosphorus cycling and effectively reduces total nitrogen and phosphorus levels in pond water [77]. This pattern suggests that the chronic elevation of NH_3_-N may have shaped the microbial community composition, potentially selecting for taxa that are tolerant to or thrive in such conditions. *Azospirillum*, which is abundant in the CG and *S.su* groups, is a nitrogen-fixing bacterium that promotes plant growth and can disrupt aquatic nutrient balance and ecosystem productivity when its abundance fluctuates [78,79]. The robust growth of *S. subulata* may have created conditions conducive to the proliferation of *Azospirillum*, a genus often associated with beneficial plant–microbe interactions.

#### 4.4.2. Genera Related to Organic Matter Degradation and Water Purification

In the three groups with added aquatic plants, *Curvibacter* dominated the water bodies of each group, indicating that the addition of aquatic plants in the fish tank may contribute to the massive reproduction of *Curvibacter*. *Curvibacter* shows potential to remove benzoate, phenylacetate, catechol, and salicylate [80]. The observed lowering of NH_3_-N levels across all planted groups provides tangible evidence for superior organic matter processing and provides a certain reference for the hypothesis that plants can improve water quality by regulating microbial communities.

Notably, *Novosphingobium* was enriched in the *A.re* group. Previous studies have reported that members of this genus may be associated with their ability to degrade compounds and promote beneficial plant root growth [81,82]. Nevertheless, our study found that the *A.re* group exhibited only moderate NH_3_-N removal and no discernible plant growth. This may suggest that, despite the enrichment of *Novosphingobium*, its beneficial functions may not have been fully realized, possibly due to the influence of other environmental factors. Thus, water quality regulation appears to be a complex process, and relying solely on the enrichment of a single microbial taxon as an indicator of improvement is likely an oversimplification. Similarly, as previous reports have shown, *Polynucleobacter* is abundant during water purification processes involving algal–bacterial biofilms [83]. Thus, its high abundance in the *W.gl* group may be closely related to the lower NH_3_-N content in the water. However, the low oxygen, low pH, and persistent decaying environment in the *W.gl* group appear to constitute a stressful condition for microbial growth. This suggests that the high abundance of *Polynucleobacter* cannot be attributed solely to water purification.

#### 4.4.3. Genus Indicating Potential Risk

In addition to these findings, an enrichment of *Asticcacaulis*—a genus associated with plant growth suppression—was detected in the CG group [84]. Notably, a study on its close relatives has been associated with gill hyperplasia and systemic hemorrhagic septicemia in turbot (*Scophthalmus maximus*) [85]. The observed enrichment of *Asticcacaulis* corresponds with the overall high NH_3_-N stress condition in the CG group, suggesting this genus may be adapted to or tolerant of such a selective environment.

#### 4.4.4. Correlation Between Pseudomonadophyta and Environmental Factors

According to these findings, except for the potential disease risk associated with *Asticcacaulis* in the CG group, Pseudomonadota enrichment primarily functioned in water purification and organic matter decomposition across treatment groups. Previous research indicates that Pseudomonadota’s role in nitrification is typically negatively correlated with ammonia nitrogen levels in aquatic systems [86]. In contrast, our study found a positive correlation between NH_3_-N concentrations and Pseudomonadota abundance. This divergence may be attributed to the suboptimal pH conditions in our experiment, which can impair bacterial metabolic efficiency [87]. These contrasting results precisely illustrate that the function of the microbial community and the water quality environment do not follow a simple one-to-one correspondence.

### 4.5. Differences in Dominant Genera Among Groups Within the Bacteroidota Phylum

Bacteroidota represented the second most abundant phylum across all treatment groups. Bacteroidota play crucial roles in organic matter degradation and nutrient cycling within biofloc systems, specializing in degrading complex organic materials, particularly polysaccharides and hydrocarbons [88]. This highlights their essential function in maintaining water quality, promoting system stability, and facilitating functional differentiation within ecosystems [89].

The genus *Sediminibacterium*, which was enriched in the CG group, is a key genus responsible for heterotrophic nitrification and aerobic denitrification [90]. The high concentrations of NH_3_-N and DO in the CG group may have contributed to its high abundance in the water.

The addition of *S. subulata* led to a high abundance of *Flavobacterium* in the water of the *S.su* group, which has been studied in previous studies and is commonly encountered in aquaculture systems, containing several opportunistic pathogens such as *F. columnare* and *F. branchiophilum* that pose threats to fish health [91]. However, no significant infection by *Flavobacterium* was observed in betta fish of the *S.su* group throughout the experimental cycle; however, studies have also indicated beneficial roles of *Flavobacterium* in maintaining plant root health [92]. Furthermore, a high abundance of *Flavobacterium* in the ponds of Chinese mitten crab (*Eriocheir sinensis*) has been correlated with denitrification [93]. Thus, this suggests that its enrichment in the *S.su* group is likely linked to advantageous traits.

The role of Bacteroidota across all groups appears consistently linked to ammonia nitrogen removal and organic matter decomposition. Existing research has shown that the abundance of Bacteroidota in pond sediments is negatively correlated with NH_3_-N concentrations [94,95]. However, our findings revealed a positive correlation between total NH_3_-N levels and Bacteroidota abundance, contradicting previous conclusions. This discrepancy may stem from differences in carbon source types and nutritional strategies [89]. These results also appear to reveal that the relationship between the microbial community and environmental factors cannot be explained by a singular inference.

### 4.6. The Function of Dominant Genus in the Verrucomicrobiota Phylum

Verrucomicrobiota contributes to organic matter decomposition and promotes carbon cycling in aquatic ecosystems, while demonstrating a sensitivity to water quality changes [96,97]. A further genus-level analysis revealed that *Luteolibacter*, the dominant genus in the *A.re* group, belongs to the phylum Verrucomicrobiota, representing a potential bacterial indicator of high-quality lakes [98]. Previous research has established that an increased Verrucomicrobiota abundance can suppress NH_4_^+^-N and NO_2_^−^-N accumulation, thereby improving water quality [99]. In our study, we indeed observed that the average NH_3_-N concentration in the *A.re* group was significantly lower compared to the CG group, suggesting its potential involvement in regulating NH_3_-N in the water. Beyond this, our study also found a negative correlation between NH_3_-N levels and Verrucomicrobiota abundance. However, a comparison with other planted groups revealed that the NH_3_-N removal efficacy of the *A.re* group was less pronounced. This raises the possibility that the functionality of a microbial community may not be directly proportional to its population size, indicating that the enrichment of a single taxon may be insufficient for system-wide regulation.

### 4.7. The Function of Dominant Genus in the Actinomycetota Phylum

Actinomycetota constitute a major bacterial phylum commonly present in aquaculture systems and in the intestinal tracts of cultured organisms [100]. This group contributes to the regulation of gut environments, pathogen inhibition, and the promotion of animal growth [101,102]. As heterotrophic nitrifiers, Actinomycetota proliferate extensively in carbon-rich aquatic environments [103], where they degrade organic compounds to provide essential nutrients for microbial communities and support the maintenance of aquatic ecological balance [104]. The high diversity observed in the *W.gl* group further underscores the significant role of Actinomycetota. The dominant genus *Mycobacterium* in the *W.gl* group, which belongs to the phylum Actinomycetota, is an opportunistic pathogen that can cause the destruction of skin and mucous membranes in aquatic animals, leading to significant harm and disease [105]. This may be associated with environmental stress factors such as low oxygen, low pH, and leaf decay, promoting the enrichment of opportunistic pathogens. Furthermore, our study observed negative correlations between NH_3_-N levels and the abundances of Actinomycetota, whereas the study by Jacobs et al. found that their abundance is positively correlated with nitrogen content and strongly negatively correlated with DO [106]. This discrepancy may be associated with divergent functions across distinct bacterial taxa.

In our study, only 12 endpoint water samples were used for microbial community analysis. These initial results showed differences among aquatic plant treatments but did not capture the full ecosystem dynamics of new betta tanks, highlighting certain limitations. It serves merely as a reference for exploring aquatic plant–microbe interactions.

## 5. Conclusions

During the construction of a new betta fish tank, DO and pH are negatively correlated with time, but NH_3_-N shows a positive correlation with time. Adding aquatic plants significantly decreased the average NH_3_-N content in the betta fish tank water under the given experimental setup, with *S. subulata* showing the most pronounced effects. The addition of aquatic plants showed a certain degree of influence on betta fish behavior. Fish in the *S.su* group were observed to exhibit shorter “resting” periods and fewer “breathing on the water surface” events. However, due to the limited recording duration, these results may not fully represent the effect of the plants. Furthermore, aquatic plants also increase microbial diversity and reshape the aquarium’s microbial community. The *W.gl* group showed the highest diversity. In all groups, the dominant phyla were Pseudomonadota and Bacteroidota. The presence of aquatic plants generally promoted the enrichment of functional genera such as *Curvibacter*. Aquatic plants increase the abundance of Verrucomicrobiota, which seems beneficial for maintaining water quality. Moreover, a systematic assessment of the optimal plant dosage was not performed, and the relatively small sample size may limit the generalizability of the results. Future studies are needed to validate these findings under more controlled and varied conditions.

## Figures and Tables

**Table 1:** Behavior ethograms of betta fish.

Behavioral Categories	Behaviors	Description
Positive (considered a good welfare state)	Swimming	Variable movement patterns with diverse swimming paths and changing speeds.
Negative (considered a negative welfare state)	Breathing on the water	Surface breathing of atmospheric air.
	Hovering	Stationary floating in mid-water without substrate contact, often with maintained pectoral fin movement.
	Stereotypical swimming	Repetitive locomotion at consistent frequency and velocity within a confined area, exemplified by back-and-forth swimming; three consecutive cycles of such movement were recorded as one stereotypic swimming event.
	Resting	Complete immobility of all body parts, including fins, while maintaining physical contact with aquarium surfaces or plants.

**Table 2:** The difference in DO, pH, and NH_3_-N in all groups.

Parameters	CG	*S.su*	*A.re*	*W.gl*
DO (mg·L^−1^)	6.16 ± 0.07 ^c^	5.98 ± 0.09 ^c^	5.00 ± 0.05 ^b^	3.88 ± 0.08 ^a^
pH	7.03 ± 0.02 ^c^	7.01 ± 0.01 ^c^	6.82 ± 0.01 ^b^	6.44 ± 0.02 ^a^
NH_3_-N(mg·L^−1^)	1.00 ± 0.08 ^d^	0.14 ± 0.02 ^a^	0.69 ± 0.06 ^c^	0.39 ± 0.03 ^b^

Note: Different lowercase superscript letters within the same row indicate significant differences (*p* < 0.05).

**Table 3:** The difference in fish behaviors in all groups within 5 min.

Group	Swimming (s)	Resting (s)	Breathing on the Surface (Times)	Hovering (s)	Stereotypical Swimming (s)
CG	170.11 ± 12.88	0.00 ± 0.00 ^a^	1.39 ± 0.25 ^b^	98.44 ± 11.06	31.44 ± 5.99
*S.su*	215.36 ± 10.01	0.14 ± 0.14 ^a^	0.36 ± 0.11 ^a^	46.50 ± 8.60	37.75 ± 7.50
*A.re*	146.86 ± 13.71	48.31 ± 12.20 ^b^	0.5 ± 0.16 ^a^	92.06 ± 13.76	13.19 ± 4.30
*W.gl*	206.81 ± 10.17	0.00 ± 0.00 ^a^	0.94 ± 0.27 ^ab^	74.53 ± 10.01	15.89 ± 4.21

Note: Different lowercase superscript letters within the same column indicate significant differences (*p* < 0.05).

**Table 4:** Plant biomass and growth rate.

	*S.su*	*A.re*	*W.gl*
Weight (g)	9.57 ± 0.32 ^c^	8.10 ± 0.40 ^b^	5.63 ± 0.12 ^a^
Growth rate (%)	19.67 ± 4.18 ^c^	1.33 ± 5.24 ^b^	−30.00 ± 1.53 ^a^

Note: Different lowercase superscript letters within the same row indicate significant differences (*p* < 0.05).

**Table 5:** Differences in microbial alpha diversity in groups.

Groups	Ace	Chao1	Shannon	Simpson
CG	507.74 ± 63.77 ^a^	489.02 ± 66.55 ^a^	2.53 ± 0.17 ^a^	0.17 ± 0.01 ^ab^
*A.re*	545.08 ± 78.05 ^ab^	532.13 ± 77.95 ^a^	2.47 ± 0.25 ^a^	0.22 ± 0.04 ^b^
*S.su*	644.66 ± 32.97 ^ab^	628.36 ± 33.84 ^ab^	2.68 ± 0.21 ^a^	0.19 ± 0.03 ^ab^
*W.gl*	800.78 ± 22.88 ^b^	790.94 ± 23.92 ^b^	3.64 ± 0.15 ^b^	0.10 ± 0.02 ^a^

Note: Different lowercase superscript letters within the same column indicate significant differences (*p* < 0.05).

## Data Availability

The original contributions presented in the study are included in the article; further inquiries can be directed to the corresponding author(s).

## Supplementary Materials

The following supporting information can be downloaded at https://www.mdpi.com/article/10.3390/ani16020247/s1. Table S1: Differences in DO of culture water in four groups for betta fish during the 0–25-day period. Table S2: Differences in pH of culture water in four groups for betta fish during the 0–25-day period. Table S3: Differences in NH_3_-N of culture water in four groups for betta fish during the 0–25-day period. Table S4: Differences in swimming time of betta fish in four groups. Table S5: Differences in resting time of betta fish in four groups. Table S6: Differences in surface breathing frequency of betta fish in four groups. Table S7: Differences in stationary hovering behavior of betta fish in four groups. Table S8: Differences in stereotypical swimming of betta fish in four groups.

## Abbreviations

The following abbreviations are used in this manuscript:

DO	Dissolved Oxygen
NH_3_-N	Ammonia Nitrogen
pH	Potential of Hydrogen
