# Supplementary material for: Effects of Aquatic Plants on Water Quality, Microbial Community, and Fish Behaviors in Newly Established Betta Aquaria
Source: Animals (Basel). 2026 Jan 14;16(2):247. doi: 10.3390/ani16020247 (PMC12838058; doi:10.3390/ani16020247)
Supplement: Supplementary file 1 [file animals-16-00247-s001.zip › animals-4060396-supplementary.pdf]

Supplementary Materials:

Table S1: Differences in DO of Culture Water in Four Groups for Betta Fish During the 0–25 Day Period.

Day	CG	*S.su*	*W.gl*	*A.re*
1	7.45±0.10	5.59±0.05	3.38±0.14	5.30±0.02
2	7.28±0.09	5.28±0.22	3.35±0.23	5.23±0.17
3	7.06±0.14	5.45±0.18	3.48±0.22	5.41±0.16
4	6.67±0.09	5.04±0.17	3.17±0.22	4.78±0.25
5	6.17±0.12	5.20±0.22	3.17±0.27	5.15±0.08
6	5.99±0.06	4.94±0.13	3.05±0.20	4.88±0.09
7	6.27±0.15	5.66±0.05	3.29±0.20	5.48±0.05
8	6.39±0.12	5.62±0.07	3.41±0.45	4.94±0.11
9	6.24±0.18	5.68±0.12	3.36±0.29	5.11±0.15
10	6.22±0.11	5.73±0.04	3.62±0.20	5.30±0.21
11	5.87±0.08	5.77±0.13	3.72±0.27	4.36±0.10
12	5.72±0.09	5.49±0.44	3.19±0.29	4.72±0.24
13	6.15±0.12	5.90±0.54	3.68±0.34	5.04±0.20
14	6.16±0.27	6.38±0.43	3.95±0.44	5.43±0.13
15	6.13±0.22	6.41±0.53	4.10±0.38	5.23±0.11
16	6.33±0.19	6.55±0.64	4.53±0.29	5.67±0.13
17	6.19±0.26	6.76±0.37	4.44±0.18	4.92±0.25
18	6.12±0.24	7.31±0.28	5.16±0.24	5.24±0.48
19	5.91±0.21	6.98±0.31	4.36±0.21	4.06±0.22
20	5.77±0.05	6.77±0.45	4.20±0.15	4.85±0.09
21	5.63±0.17	6.66±0.14	4.36±0.20	4.82±0.13
22	5.96±0.08	6.54±0.21	4.34±0.15	5.16±0.32
23	5.68±0.13	6.41±0.24	4.59±0.19	4.96±0.20
24	5.34±0.35	5.52±0.51	4.45±0.22	4.52±0.13
25	5.20±0.57	5.75±0.43	4.60±0.23	4.67±0.13

Table S2: Differences in pH of Culture Water in Four Groups for Betta Fish During the 0-25 Day Period.

Day	CG	*S.su*	*W.gl*	*A.re*
1	7.23±0.06	7.12±0.01	6.81±0.03	6.96±0.04
2	7.22±0.15	6.92±0.01	6.77±0.03	6.93±0.07
3	7.16±0.01	7.03±0.02	6.73±0.02	6.89±0.04
4	7.11±0.05	7.05±0.03	6.70±0.02	6.89±0.02
5	7.09±0.01	7.10±0.03	6.61±0.04	6.98±0.03
6	7.09±0.01	7.13±0.03	6.67±0.02	6.88±0.10
7	7.41±0.08	7.18±0.03	6.56±0.06	6.94±0.01
8	7.00±0.03	6.93±0.02	6.40±0.02	6.70±0.02
9	6.74±0.03	6.94±0.01	6.41±0.02	6.82±0.06
10	6.93±0.01	6.94±0.03	6.38±0.03	6.78±0.03

11	6.94±0.03	6.93±0.01	6.40±0.03	6.73±0.01
12	7.00±0.02	6.97±0.02	6.47±0.05	6.85±0.00
13	6.99±0.02	6.90±0.01	6.41±0.04	6.75±0.04
14	7.00±0.01	6.98±0.03	6.38±0.03	6.79±0.01
15	6.94±0.02	6.94±0.01	6.45±0.07	6.79±0.05
16	6.98±0.02	6.99±0.05	6.34±0.07	6.80±0.03
17	7.01±0.03	7.00±0.08	6.27±0.06	6.77±0.01
18	6.99±0.03	7.06±0.03	6.21±0.07	6.78±0.02
19	6.92±0.06	7.02±0.01	6.41±0.04	6.83±0.02
20	7.02±0.01	7.11±0.01	6.33±0.06	6.74±0.02
21	7.00±0.04	7.09±0.00	6.30±0.08	6.76±0.02
22	7.03±0.01	7.06±0.01	6.33±0.09	6.80±0.00
23	6.94±0.03	7.01±0.01	6.32±0.08	6.84±0.00
24	6.90±0.04	6.91±0.02	6.25±0.09	6.76±0.01
25	6.99±0.04	7.00±0.03	6.16±0.10	6.77±0.01

Table S3: Differences in NH_3-N of Culture Water in Four Groups for Betta fish During the 0–25 Day Period.

Day	CG	*S.su*	*W.gl*	*A.re*
1	0.32±0.02	0.05±0.02	0.09±0.05	0.25±0.05
2	0.46±0.02	0.09±0.02[a]	0.27±0.04	0.28±0.04
3	0.53±0.01	0.11±0.00	0.33±0.06	0.35±0.06
4	0.49±0.05	0.05±0.01	0.28±0.03	0.32±0.03
5	0.86±0.04	0.20±0.07	0.45±0.09	0.61±0.09
6	0.82±0.02	0.11±0.02	0.29±0.04	0.53±0.04
7	0.84±0.04	0.08±0.02	0.35±0.04	0.63±0.04
10	1.04±0.08	0.06±0.01	0.34±0.05	0.64±0.05
13	1.06±0.12	0.08±0.02	0.31±0.03	0.76±0.03
16	1.39±0.06	0.09±0.01	0.40±0.06	1.13±0.06
19	1.42±0.10	0.33±0.04	0.39±0.01	1.06±0.11
22	1.64±0.04	0.21±0.02	0.60±0.22	1.03±0.22
25	2.09±0.07	0.32±0.01	0.95±0.30	1.29±0.30

Table S4: Differences in Swimming Time of Betta Fish in Four Groups.

Day	CG	*A.re*	*S.su*	*W.gl*
1	197.67±35.09	51.67±10.27	182.67±40.44	225.33±32.77
2	128.00±30.00	129.00±55.87	129.67±41.24	195.33±25.71
3	216.67±37.67	112.33±39.83	237.33±9.33	218.33±27.72
4	219.00±13.80	223.00±68.04	259.33±22.58	215.67±13.35
5	180.00±41.28	199.00±58.20	231.00±18.88	189.33±39.28
6	168.00±63.67	187.67±27.67	257.67±39.86	246.00±31.48
9	158.33±66.74	191.00±41.40	173.33±52.92	117.33±38.71
12	225.33±5.70	136.33±29.49	210.67±15.96	175.00±49.40
15	99.33±46.68	197.00±43.55	237.00±30.75	251.33±48.67

18	167.67±26.49	105.33±58.98	248.00±10.50	247.67±15.59
21	141.00±82.59	116.00±45.30	176.67±41.91	182.00±25.38
24	140.33±50.61	114.00±36.12	241.00±11.59	218.33±36.36

Table S5: Differences in Resting Time of Betta Fish in Four Groups.

Day	CG	*A.re*	*S.su*	*W.gl*
1	0.00±0.00	39.00±39.00	0.00±0.00	0.00±0.00
2	0.00±0.00	26.67±26.67	0.00±0.00	0.00±0.00
3	0.00±0.00	88.33±22.42	1.67±1.67	0.00±0.00
4	0.00±0.00	4.33±4.33	0.00±0.00	0.00±0.00
5	0.00±0.00	5.00±5.00	0.00±0.00	0.00±0.00
6	0.00±0.00	58.33±12.57	0.00±0.00	0.00±0.00
9	0.00±0.00	11.33±5.93	0.00±0.00	0.00±0.00
12	0.00±0.00	18.67±18.67	0.00±0.00	0.00±0.00
15	0.00±0.00	5.00±5.00	0.00±0.00	0.00±0.00
18	0.00±0.00	187.67±64.95	0.00±0.00	0.00±0.00
21	0.00±0.00	86.33±76.0.008	0.00±0.00	0.00±0.00
24	0.00±0.00	49.00±49.00	0.00±0.00	0.00±0.00

Table S6: Differences in Surface Breathing Frequency of Betta Fish in Four Groups

Day	CG	*A.re*	*S.su*	*W.gl*
1	0.33±0.33	0.00±0.00	0.00±0.00	0.00±0.00
2	0.33±0.33	0.00±0.00	0.33±0.33	0.00±0.00
3	1.67±0.67	0.00±0.00	0.33±0.33	2.00±0.58
4	0.00±0.00	0.67±0.67	0.67±0.67	0.00±0.00
5	1.33±0.33	0.33±0.33	0.00±0.00	2.67±1.33
6	1.67±0.88	2.00±1.53	0.67±0.67	1.67±1.67
9	3.67±1.20	0.67±0.33	0.00±0.00	0.00±0.00
12	2.00±1.53	1.00±0.58	0.00±0.00	2.67±1.45
15	2.00±0.58	0.67±0.33	1.00±0.58	0.00±0.00
18	1.67±0.33	0.00±0.00	0.00±0.00	0.33±0.33
21	0.67±0.67	0.33±0.33	1.00±0.58	1.00±1.00
24	1.33±1.33	0.33±0.33	0.33±0.33	1.73±0.87

Table S7: Differences in Stationary Hovering Behavior of Betta Fish in Four Groups

Day	CG	*A.re*	*S.su*	*W.gl*
1	97.67±36.33	209.33±38.52	103.00±53.5	32.67±16.18
2	109.00±3.79	138.00±63.11	123.33±64.0	67.33±18.02
3	63.00±25.70	99.33±50.66	48.00±10.69	71.00±29.46
4	65.67±22.41	72.67±70.18	36.67±22.64	65.67±17.95
5	54.67±8.29	91.33±52.54	20.00±10.41	107.33±39.54
6	42.67±12.91	64.33±40.48	24.67±4.48	76.67±60.47
9	115.33±48.72	87.67±45.80	26.33±13.69	157.33±55.17

12	82.00±38.14	39.00±24.09	33.33±30.87	43.33±21.31
15	175.33±32.63	95.67±48.52	38.00±18.82	44.33±44.33
18	109.67±12.88	7.00±7.00	39.33±11.46	49.00±15.87
21	159.00±82.59	87.67±58.65	41.67±15.65	103.00±24.58
24	107.33±46.45	112.67±9.56	26.67±8.82	76.67±33.75

Table S8: Differences in Stereotypical Swimming of Betta Fish in Four Groups

Day	CG	*A.re*	*S.su*	*W.gl*
1	4.67±4.67	0.00±0.00	14.33±14.33	8.67±8.67
2	63.00±26.54	6.33±6.33	47.00±30.81	37.33±15.59
3	20.33±13.54	0.00±0.00	13.00±6.56	10.67±10.67
4	15.33±15.33	0.00±0.00	4.00±4.00	18.67±9.40
5	65.33±35.26	4.67±2.60	49.00±17.21	3.33±3.33
6	32.00±18.61	41.00±23.97	64.67±13.69	48.33±34.44
9	26.33±26.33	10.00±10.00	100.33±41.95	25.33±25.33
12	50.00±25.53	54.67±30.91	9.00±9.00	10.67±10.67
15	25.33±14.72	7.33±7.33	25.00±11.93	4.33±4.33
18	22.67±13.68	0.00±0.00	12.67±12.67	3.33±3.33
21	0.00±0.00	10.00±10.00	81.67±48.24	15.00±8.66
24	52.33±21.71	24.33±17.38	32.33±16.19	5.00±2.89
